# Supplementary material for: ArtSeg—Artifact segmentation and removal in brightfield cell microscopy images without manual pixel-level annotations
Source: Sci Rep. 2022 Jul 6;12:11404. doi: 10.1038/s41598-022-14703-y (PMC9259686; doi:10.1038/s41598-022-14703-y)
Supplement: Supplementary file 1 — Supplementary Information. [file 41598_2022_14703_MOESM1_ESM.pdf]

Supplementary Material

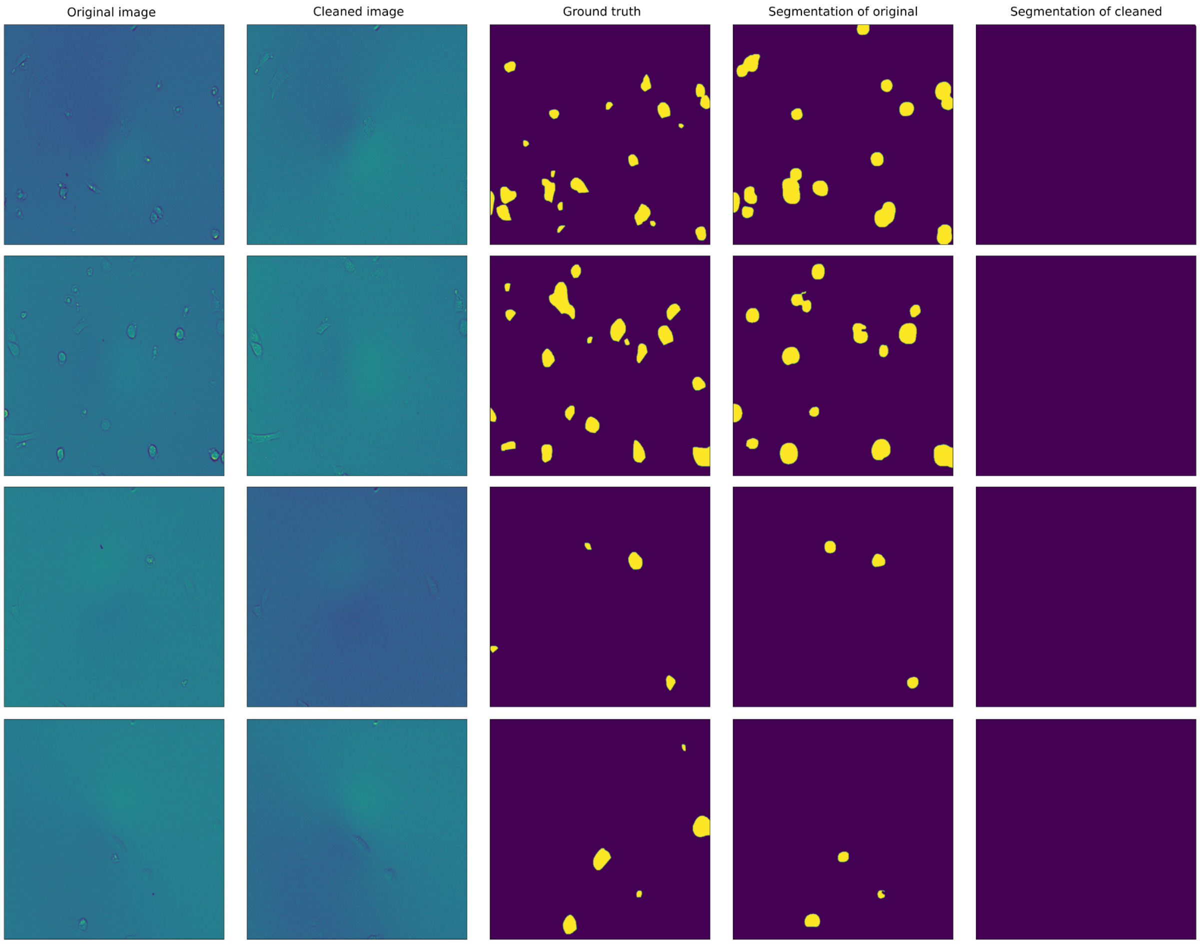

Supplementary Figure 1: Example of artifact-free generated images. **Left to right:** original image with artifacts, artifact-free images generated from the image in the left column, human-generated ground truth, model segmentation of the original image in the leftmost column, model segmentation of the artifact-free image in the second left column.

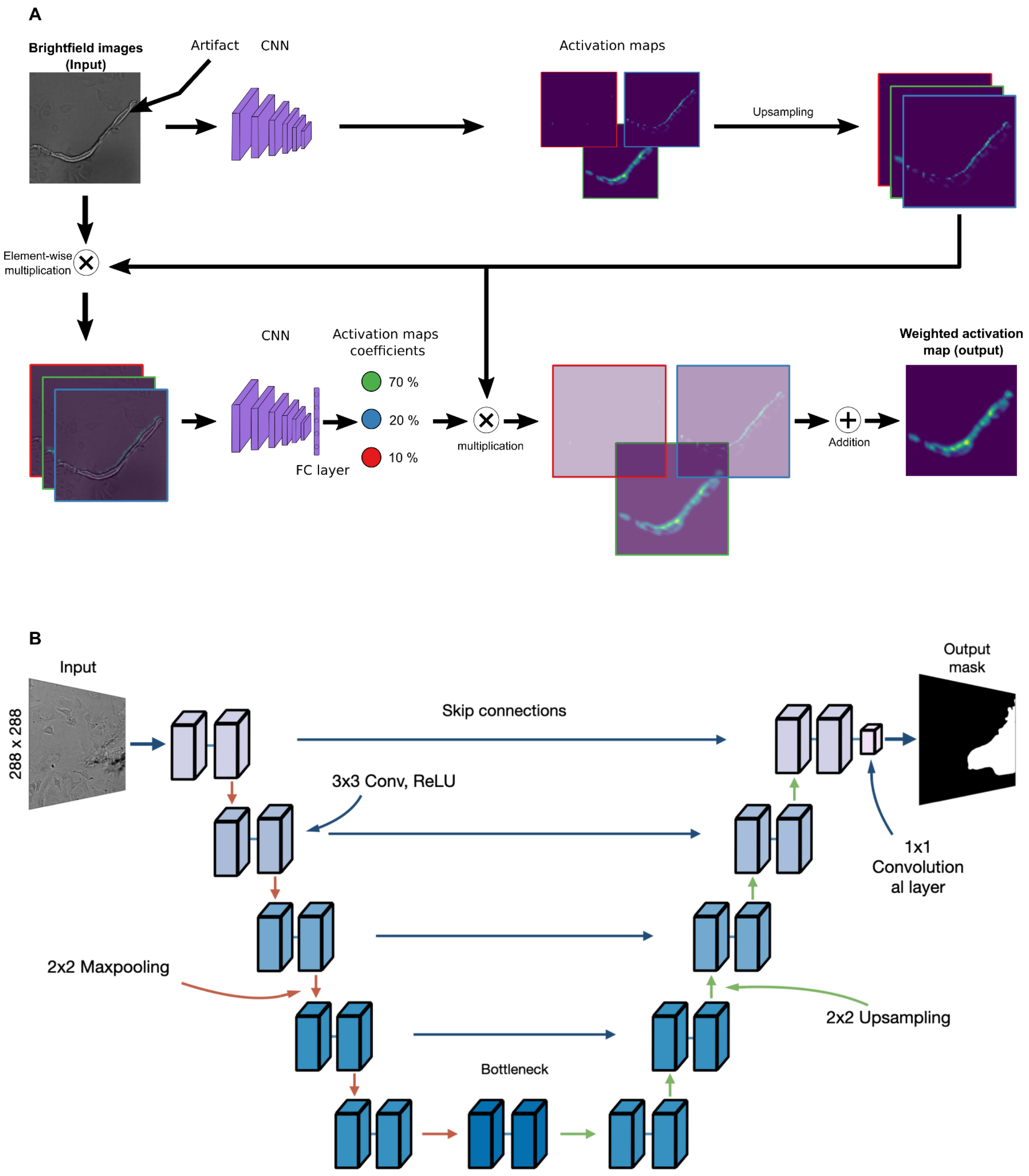

Supplementary Figure 2: Zoomed components of the pipeline. **A**) ScoreCAM[1] algorithm which is used to generate the ground truth to train U-Net during training, and used to determine whether there are any artifact objects in the image during inference. **A**) U-Net model used for object segmentation.

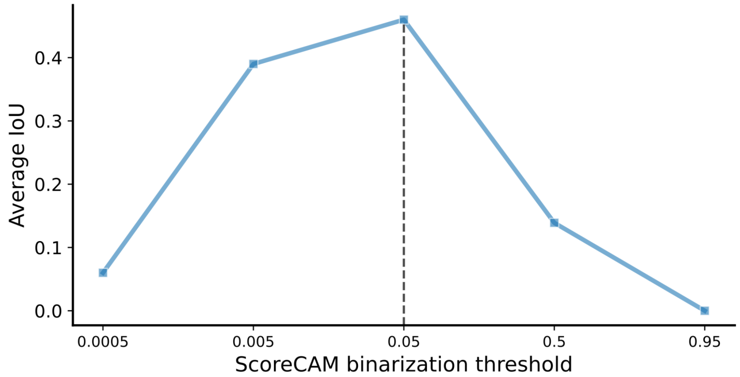

Supplementary figure 3: ScoreCAM binarization threshold selection. ScoreCAM-U-Net was trained using different thresholds (x-axis) for binarizing the results of ScoreCAM. The threshold with the best IoU score on the validation set (y-axis) was selected (black-dashed line).

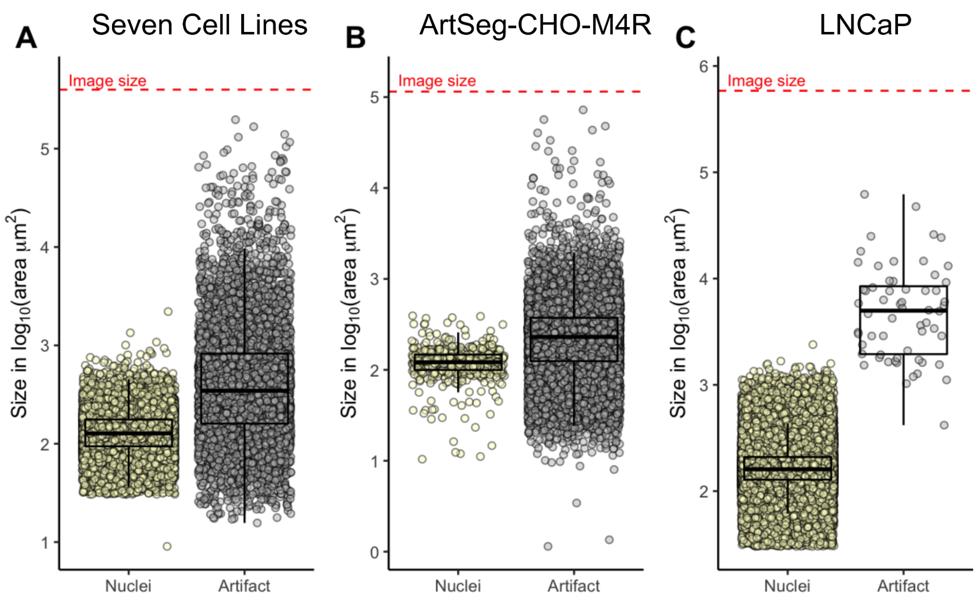

Supplementary Figure 4: Artifacts are in different sizes. Size (mm^2 in log$_{10}$ scale, y-axis) of nuclei and artifact objects (colors, x-axis) in seven cell lines **(A)**, ArtSeg-CHO-M4R **(B)**, and LNCaP datasets **(C)**. Boxes: 25th, 50th and 75th percentile; whiskers: 1.5x from the interquartile range; circles: individual artifact or nucleus. Dashed red line: size of the whole image.

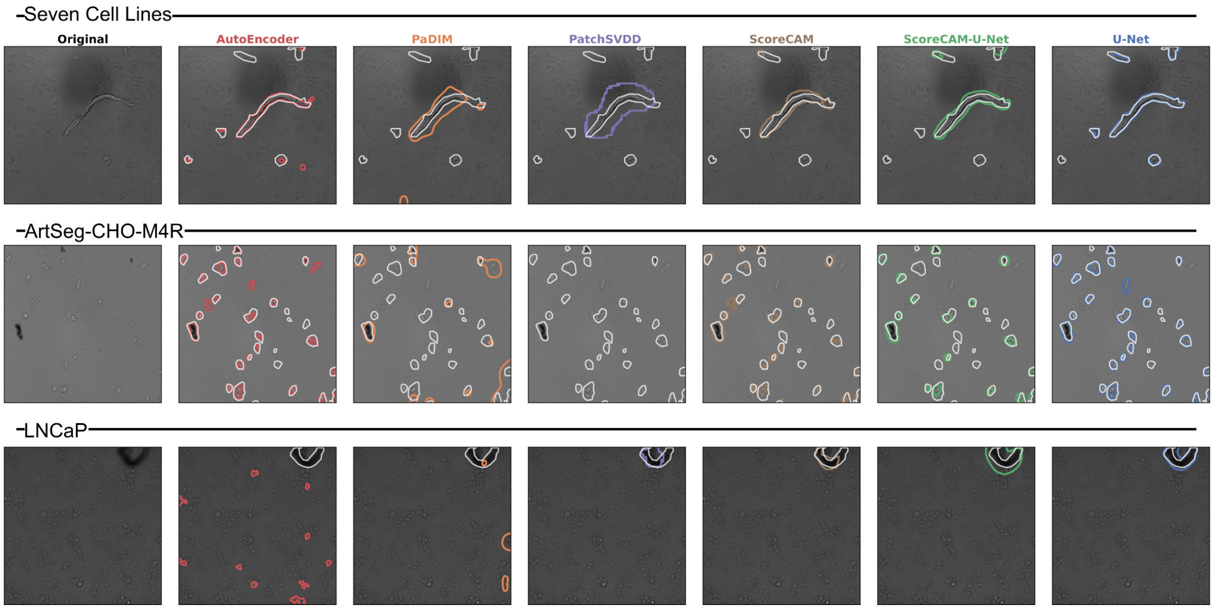

Supplementary Figure 5: Artifact segmentation examples for all models (colors) in seven cell lines, LNCaP, and ArtSeg-CHO-M4R datasets (rows). Examples of brightfield images and the corresponding artifact segmentation of all models (columns, colors) and datasets (rows; separated by lines and dataset names). White contour: true artifact boundaries; colored contours: artifact segmentation boundaries of the corresponding model.

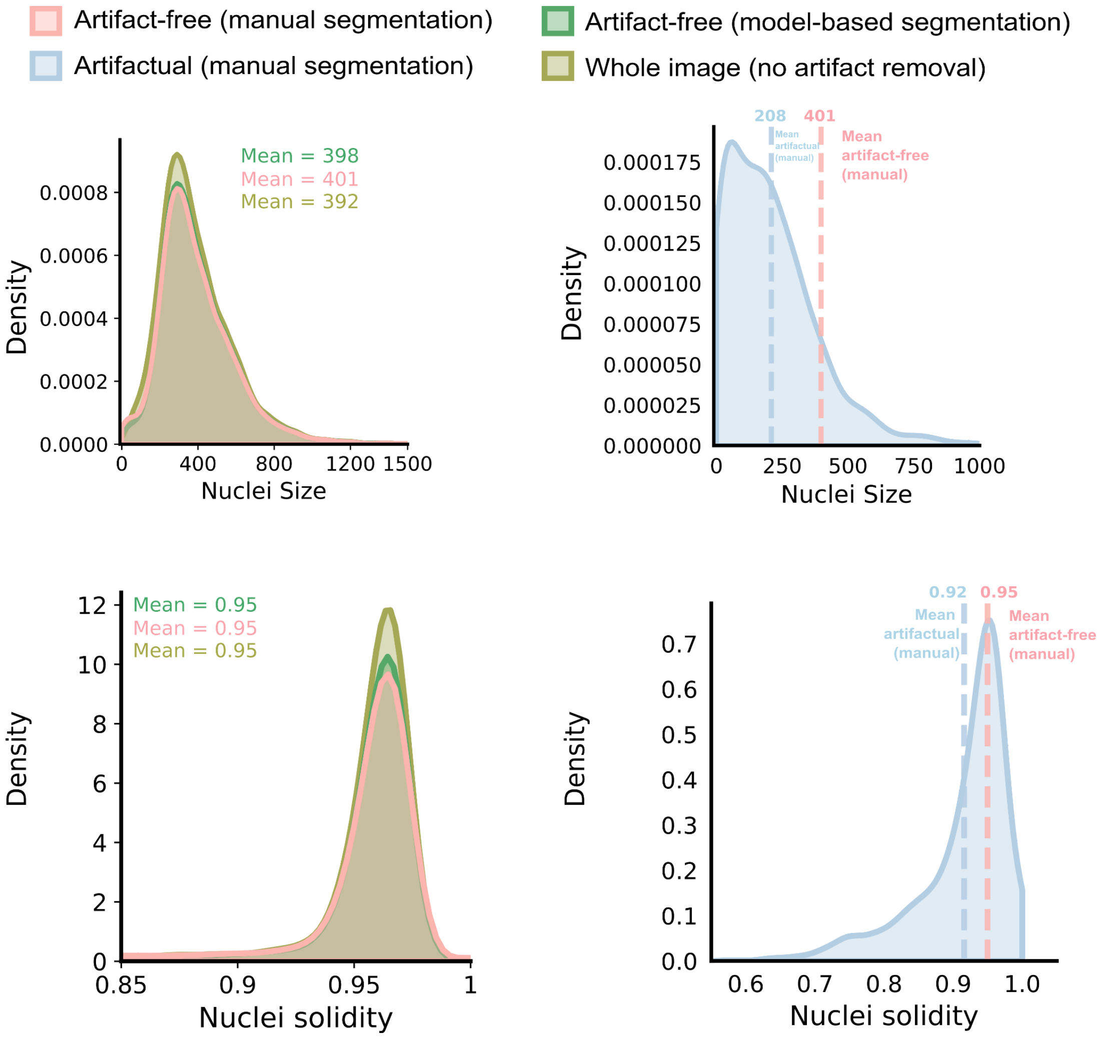

Supplementary Figure 6: Impact of artifacts and artifact removal on downstream analyses. Density (y-axis) plots of segmented nucleus size in pixels (top, x-axis) and solidity (bottom, x-axis) in the seven cell lines dataset for different areas of the image (colors). Metrics are calculated for different areas of the images (colors); artifactual: artifactual areas in the images; artifact-free: area in the images other

than the artifactual areas. Artifacts are detected in two ways; manual: the detection of artifacts is performed manually; model-based: the ScoreCAM-U-Net model is used to detect the artifacts. No artifact removal: the metrics are calculated without removing artifacts. Dashed lines: mean size in pixels and solidity of segmented nucleus in the artifactual and artifact-free regions(different colors).

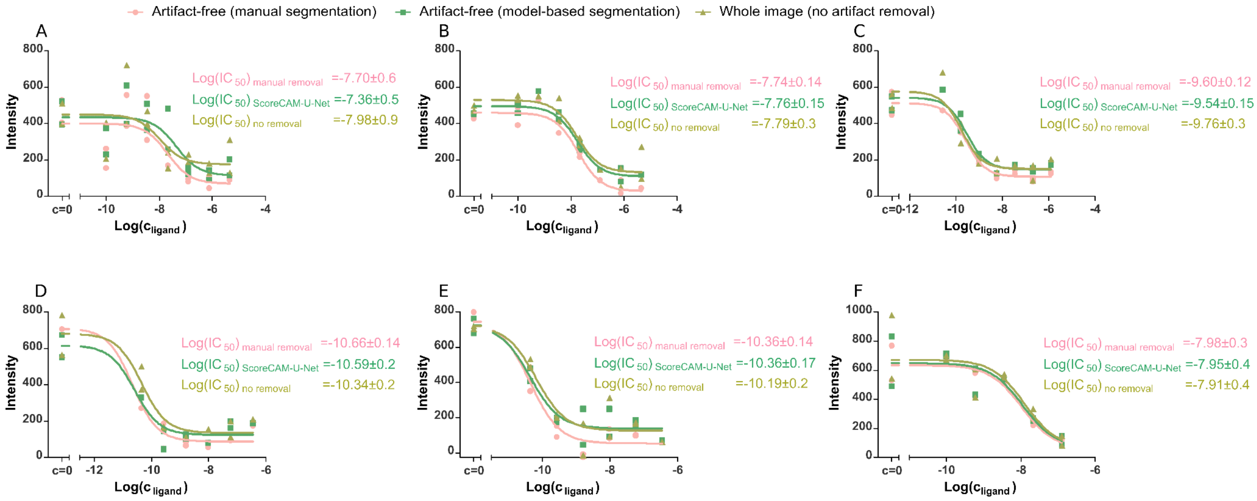

Supplementary Figure 7: Cell fluorescence intensity dependence on M4 receptor ligand concentration determined with live-cell fluorescence microscopy at the presence of 2 nM UR-CG072. Displacement curves of three different ligands are shown: UNSW-MK259 (**A, B** and **F**), atropine (**D** and **E**) and UR-SK75 **(C)**. Three different artifact removal methods at the image analysis stage are compared (colors): manual artifact segmentation, ScoreCAM-U-Net segmentation and no artifact removal. For each combination of ligand and artifact removal method a regression analysis is performed with Hill equation (Hill coefficient fixed at 1) with the best fits shown as continuous lines. Each displacement curve was measured in duplicates with each data point representing the average fluorescence intensity of cells in each well.

Supplementary Table 1: U-Net model architecture[2]. Output Filters are the number of trained filters, which correspond to the number of feature maps. Concatenation (Down x) means concatenating the output of block down x with this layer. Output Dimensions denotes the height and width of the output feature maps.

U-Net block	Layers	Output Dimensions	Output Filters
**Input**	Input	288x288	64
**Down 1**	Convolution 3x3, ReLU	288x288	64
	Convolution 3x3, ReLU	288x288	64
	Max pooling 2x2	144x144	64
**Down 2**	Convolution 3x3, ReLU	144x144	64
	Convolution 3x3, ReLU	144x144	64
	Max pooling 2x2	72x72	64
**Down 3**	Convolution 3x3, ReLU	72x72	64
	Convolution 3x3, ReLU	72x72	64
	Max pooling 2x2	36x36	64
**Down 4**	Convolution 3x3, ReLU	36x36	64
	Convolution 3x3, ReLU	36x36	64
	Max pooling 2x2	18x18	64
**Down 5**	Convolution 3x3, ReLU	18x18	64
	Convolution 3x3, ReLU	18x18	64
	Max pooling 2x2	9x9	64
**Bottleneck**	Convolution 3x3, ReLU	9x9	64
	Convolution 3x3, ReLU	9x9	64
**Up 1**	Upsampling 2x2	18x18	64
	Concatenation (Down 5)	18x18	128
	Convolution 3x3, ReLU	18x18	64

	Convolution 3x3, ReLU	18x18	64
**Up 2**	Upsampling 2x2	36x36	64
	Concatenation(Down 4)	36x36	128
	Convolution 3x3, ReLU	36x36	64
	Convolution 3x3, ReLU	36x36	64
**Up 3**	Upsampling 2x2	72x72	64
	Concatenation(Down 3)	72x72	128
	Convolution 3x3, ReLU	72x72	64
	Convolution 3x3, ReLU	72x72	64
**Up 4**	Upsampling 2x2	144x144	64
	Concatenation(Down 2)	144x144	128
	Convolution 3x3, ReLU	144x144	64
	Convolution 3x3, ReLU	144x144	64
**Up 5**	Upsampling 2x2	288x288	64
	Concatenation(Down 1)	288x288	128
	Convolution 3x3, ReLU	288x288	64
	Convolution 3x3, ReLU	288x288	64
**Output**	Convolution 3x3, sigmoid	288x288	1

Supplementary Table 2: Intersection over union of all models in the seven cell lines dataset after removing predicted objects smaller than different cutoff sizes

**Object size cutoff(pixels)**	**AE**	**PaDIM**	**Patch_SVDD**	**ScoreCAM**	**ScoreCAM-U-Net**	**U-Net**
20	21.1	33.4	27.9	23.4	52	72.9
50	21.2	33.4	27.9	23.5	52.1	72.9
100	21.5	33.4	27.9	23.5	52.1	72.9
500	24.8	33.4	27.9	23.9	53.1	72.9
1000	28.6	33.3	27.9	24.2	53.6	73
2000	33.3	33.2	27.9	24.2	54.1	73.2

Supplementary Table 3: Intersection over union of all models in the ArtSeg-CHO-M4R dataset after removing predicted objects smaller than different cutoff sizes

Object size cutoff (pixels)	AE	PaDIM	Patch_SVDD	ScoreCAM	ScoreCAM-U-Net	U-Net
20	26.8	13.4	3.4	27.3	23.1	44.4
50	26.8	13.4	3.4	27.3	23.1	44.4
100	26.9	13.4	3.4	27.3	23.1	44.4
500	26.9	13.3	3.4	26.5	22.9	43.7
1000	24.2	13.1	3.4	24.8	22.3	42
2000	18	12.3	3.4	20.3	20.2	35

Supplementary Table 4: Intersection over union of all models in the LNCaP dataset after removing predicted objects smaller than different cutoff sizes

Object size cutoff (pixels)	AE	PaDIM	Patch_SVDD	ScoreCAM	ScoreCAM-U-Net	U-Net
20	0	9.6	2.8	28.2	44.8	62.9
50	0	9.6	2.8	28.3	44.8	62.9
100	0	9.6	2.8	28.3	44.8	62.9
500	0	9.7	2.8	29.1	44.7	62.9
1000	0	9.3	2.8	28.5	44.7	62.9
2000	0	9.6	2.8	29.4	44.7	63.8

Supplementary Table 5: Segmentation and image-level detection/classification results in seven cell lines, ArtSeg-CHO-M4R, and LNCaP datasets. PW: pixel-wise; IoU: intersection over union.

Dataset	Metric	U-Net	ScoreCAM-U-Net	ScoreCAM	AE	PADIM	Patch_SVDD
Seven Cell Lines	Classification F1-Score	89.7	93.2	93.2	66.7	90.1	63.9
	Segmentation PW-F1	84.4	66.2	47.2	39.7	50	28.3
	Segmentation PW-precision	86	74.4	42	32.4	59.7	39.6
	Segmentation PW-recall	82.8	61	54.6	51.4	43.1	23.9
	Segmentation IoU	72.9	49.5	31.1	24.8	33.4	17.1
ArtSeg-CHO-M4R	Classification F1-Score	80.8	90	90	66.7	72.7	23.6
	Segmentation PW-F1	62	34.9	40.1	42.4	22.6	3.1
	Segmentation PW-precision	79	30.3	58.1	47.3	36.1	40.4
	Segmentation PW-recall	51.4	41.5	30.9	38.5	19.4	26.6
	Segmentation IoU	45	21.3	25.1	26.9	12.8	1.6
LNCaP	Classification F1-Score	99.4	93.7	93.7	69.1	80	59.1
	Segmentation PW-F1	79.3	55.7	52	0	17.7	11.6
	Segmentation PW-precision	75	50.5	75.2	0	17.8	9.9
	Segmentation PW-recall	84.4	79.5	40.9	0	17.6	41.6
	Segmentation IoU	65.7	39.9	35.2	0	9.7	6.9

Reference

1. Wang, H., Wang, Z., Du, M. & Yang, F. Score-CAM: Score-weighted visual explanations for convolutional neural networks. *Proceedings of the* (2020).
2. Fishman, D. *et al.* Practical segmentation of nuclei in brightfield cell images with neural networks trained on fluorescently labelled samples. *J. Microsc.* **284**, 12–24 (2021).

Appendix A

Score-CAM-U-Net can be defined as:

$$Y_i = f(t(L^{cl}_{Score-CAM}(X_i))) \tag{1}$$

In equation (1), $X_i(H, W, C)$, $Y_i(H, W, C)$ are the input and the output tensors, respectively, where H, W are spatial dimensions and C is channel dimension; $t(.)$ denotes the thresholding operation so that the tensor's scalar values are 0 or 1; f is the ConvNet operator; and $L^{c}_{Score-CAM}$ is the Score-CAM algorithm.

Consider a convolutional layer l in a model g, given a class of interest $cl.$ g takes input x and outpupts a probability that this input is being classified as $cl.$ Score-CAM can be defined as follows[1] :

$$L^{c}_{Score-CAM} = ReLU\,(\Sigma_k\, \alpha^{cl}_k\, A^k_l\,) \tag{2}$$

Where A^k_l is activation map of the k^{th}channel in layer l and

$$\alpha^{cl}_k = CIC(A^k_l) \tag{3}$$

Where CIC is the Channel-wise Increase of Confidence[1] of activation map A^k_l.

$f(.)$ is the U-Net segmentation model which consists of a series of blocks having convolution, pooling, and concatenation operations. The details of the model are in Supplementary Table 1.
